# Determination of hand and palm area as a ratio of body surface area in Indian population

**DOI:** 10.4103/0970-0358.63962

**Published:** 2010

**Authors:** Pawan Agarwal, Sashikant Sahu

**Affiliations:** Plastic Surgery Unit, Department of Surgery, N.S.C.B. Government Medical College, Jabalpur, India

**Keywords:** Body surface area, hand area, palm area

## Abstract

**Background:**

Accurate estimation of body surface area (BSA) burn is important. In small and patchy burns, the patient's hand is used to estimate percentage of burn which is traditionally considered as 1%. There is discrepancy about what percentage of TBSA is constituted by the palm and hand. Therefore, this study was designed to determine correctly the TBSA represented by the palmar surface of the entire hand and palm in the Indian population.

**Material and Methods:**

300 healthy adult (male and female) and 300 healthy children (male and female) were included in the study. TBSA was calculated using DuBois formula and hand and palm surface area was calculated using hand tracing on plain paper. The hand/palm percentage of BSA (ratio) was determined by dividing hand/palm surface area by total BSA.

**Results:**

The mean hand and palm ratio for adults was 0.92% and 0.50%, respectively. The mean hand and palm ratio in children was 1.06% and 0.632%, respectively.

**Conclusion:**

The hand area (palm plus digits) is more closely represented to 1% of TBSA in Indian population.

## INTRODUCTION

Thermal burns and related injuries are a major cause of death and disability. The single most important factor in predicting burn-related mortality, need for specialized care, likelihood of complications, treatment plans, including initial resuscitation and subsequent nutritional requirement the size of burn.[1] Therefore, accurate estimation of size of burn is important. An additional benefit of accurate estimation of body surface area (BSA) burn is to compare the treatment outcomes of patients between different institutions.[2]

There are several methods for assessment of burn size. These include the 'rule of nine' and Lund and Browder chart.[3] Sometimes these methods are used in combination. The 'rule of nine' is a convenient and rapid method of estimating the extent of BSA burned. It is fairly accurate in adults and small burns, but it is not very accurate in case of patchy and pediatric burns.[1] The more accurate method of measuring the extent of total BSA burn is the Lund and Browder chart, which subdivides body areas into segments and assigns a proportionate percentage of body surfaces to each area based on the age. It compensates for the variation in body shape with age, and therefore, can give an accurate assessment of burn areas in children also. However, it is not a convenient method and charts are not available particularly outside the hospital environment for the initial assessment. Another method used in small and patchy burn is to use the patient's hand to estimate percentage BSA (BSA) of burn which is traditionally considered as 1%. Exactly what constitutes 'the palm of the hand' and how large an area it is depends on whether you follow advanced trauma life support (ATLS) teaching, UK teaching or use a 'Lund and Browder' chart. ATLS teaches that the area of the palm (hand minus digits) is equal to 1% of BSA.[4] In the UK, it is generally thought that the area from the distal wrist crease to the tips of the fingers (Palm Plus digits) is equivalent to 1% BSA.[5] A standard Lund and Browder chart shows that the area from the wrist to the tip of the fingers as 1.5%,[3] thus there is discrepancy about what percentage of TBSA is constituted by the palm and hand. Therefore, this study was designed to determine correctly the TBSA represented by the palmar surface of the entire hand and palm in Indian population.

## MATERIAL AND METHODS

This study was conducted in the plastic surgery unit, Department of Surgery, Netaji Subhash Chandra Bose Medical College, Jabalpur over a period of one year. 300 healthy adult and 300 healthy children of both sexes and from varying social and cultural backgrounds, representing the general population, were included in the study. Subjects were numbered and age, gender, height, weight and dominant hand were noted. The dominant hand tracing was obtained by asking the volunteer to keep the hand flat on a plain sheet of paper, keeping the fingers together and thumb lying comfortably against the radial aspect of hand and index finger. A tracing was made using pencil. The tracing started from tip of the radial styloid and passing all around the hand ended at the tip of ulnar styloid. The area of tracing was closed with a straight line drawn between the two styloid tips and termed the interstylon. The percentage of hand area was calculated by following method.[6] The length of the hand was measured from the midpoint of the interstylon to the distal tip of the middle finger. The length of the palm was measured from the midpoint of the interstylon to the palmar digital crease of middle finger. Additionally the width of the hand was measured from the ulnar aspect at the palmar digital crease of small finger to the point where the thumb diverged from the index finger. Width of the hand is multiplied with the length of hand and palm to gives hand and palm area, respectively.

**Figure 1:**
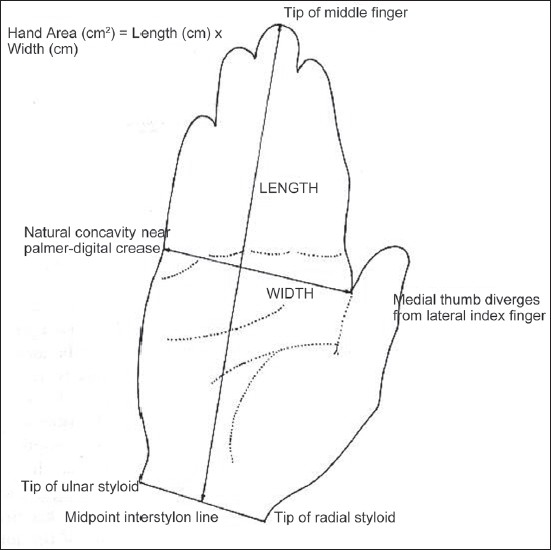
The BSA calculated using DuBois formula

The BSA was calculated using DuBois formula BSA= 71.84 W^.425^ H^.725^,[7] where W is weight in kg and H is height in cm [Figure 1].

The hand's percentage of BSA (hand ratio) was determined by dividing hand area by total BSA. Similarly the palm's percentage of BSA (palm ratio) was determined by dividing the palm area by total BSA. The mean values of TBSA, palm ratio, hand ratio were tested using the Student *t* test. All means were expressed as mean ± standard deviation. The critical level of significance of the results was considered at 0.05 levels i.e. *P* < 0.05 was considered significant.

## RESULTS

In this study 300 adults and 300 children at random were selected in order to determine hand area and palm area ratio as compared to BSA in Indian population. The age group in pediatric patients was 2-17 years and in adults it was 18-60 years. The mean TBSA for male and female was 1.59 m^2^, and 1.43 m^2^, respectively and for combined male and female was 1.51 m^2^. These values were less as compared to western population which is 1.9 m^2^, 1.6 m^2^ and 1.7 m^2^ for male, female and combined, respectively.[8] This may be due to short stature of Indian adults. The mean TBSA for male child, female child and combined were 0.810 m^2^, 0.800 m^2^ and 0.805 m^2^, respectively [Table 1].

**Table 1:** Mean TBSA (m^2^) in children and adult

*Group*	*Sex*	*N*	*Mean*	*Std. deviation*
Children	Male	150	0.81034	0.201127
	Female	150	0.80092	0.190835
	Total	300	0.80563	0.195777
Adult	Male	150	1.59453	0.121394
	Female	150	1.43995	0.140370
	Total	300	1.51724	0.152170

In our study the mean hand area in adult male was 146.50 cm^2^, while in female it was 132.42 cm^2^. The mean hand area for combined male and female was 139.46 cm^2^. There is statistically significant difference in the hand area between male and female (*P* < 0.0001) [Table 2].

**Table 2:** Hand area (cm^2^) and palm area (cm^2^) in studied cases

*Group*	*Sex*	*N*	*Mean hand area*	*Mean palm area*
Children	Male	150	86.517 ± 22.7072	51.700 ± 13.5030
	Female	150	84.774 ± 19.4205	49.650 ± 11.5876
	Total	300	85.646 ± 21.1106	50.675 ± 12.6026
Adult	Male	150	146.500 ± 15.0994	77.853 ± 10.2858
	Female	150	132.424 ± 14.1279	73.659 ± 9.1420
	Total	300	139.462 ± 75.756	75.756 ± 9.9389

The mean palm area in adult male was 77.85 cm^2^ and in adult female was 73.65 cm^2^. The mean palm area for combined male and female was 75.75 cm^2^. There is statistically significant difference in palm area between male and female (*P* < 0.001) [Table 2].

The hand ratio in adult male and female was 0.92% and 0.92%, respectively, and the mean hand ratio for combined male and female was 0.92%. There was no significant difference between the hand ratio in adult male and female (*P* > 0.05) [Table 3].

**Table 3:** Hand ratio and palm ratio in studied cases

*Group*	*Sex*	*N*	*Hand ratio (%)*	*Palm ratio (%)*
Children	Male	150	1.06722 ± 0.111630	0.63978 ± 0.085817
	Female	150	1.06397 ± 0.110189	0.62539 ± 0.082414
	Total	300	1.06560 ± 0.110738	0.63259 ± 0.084301
Adult	Male	150	0.92228 ± 0.102072	0.49082 ± 0.071324
	Female	150	0.92164 ± 0.072727	0.51321 ± 0.056781
	Total	300	0.92196 ± 0.088475	0.50201 ± 0.065325

The palm ratio in adults male and female was 0.49% and 0.51%, respectively. The mean palm ratio for combined male and female was 0.50%. There was significant difference in palm ratio been male and female (*P* < 0.01) [Table 3].

In our study the mean hand area in male and female children were 86.51 cm^2^ and 84.77 cm^2^ respectively. Combined value in male and female child was 85.64 cm^2^. The male and female values are comparable with no significant difference (*P* > 0.05) [Table 2].

The mean palm area in male and female child was 51.70 cm^2^ and 49.65 cm^2^. Combined value in male and female child was 50.67 cm^2^. The mean palm area of male and female child is comparable with no statistically significant difference (*P* > 0.05) [Table 2].

The hand ratio in male and female children was 1.06% and 1.06%, respectively. The combined value in male and female child was 1.06%. This study also reveals that the hand ratio in case of male and female children was comparable with (*P* > 0.05) [Table 3].

The palm ratio in male and female children was 0.63% and 0.62%, respectively. The combined value for male and female was 0.632%. The palm ratio between male and female children was comparable with no significant difference (*P* > 0.01) [Table 3].

## DISCUSSION

There is wide debate on what percentage constitutes hand and palm area and existing literature gives the value for western population,[9‐12] and hence, it is important to know the hand and palm area in the Indian population.

In our study, the adult hand ratio was 0.92% compared to 0.77% by Perry[9] and 0.78% by Amirsheybani.[6] The average area of hand was 0.81% in males and 0.67% in females by Rossiter.[10] In our study, the average area of hand was 0.92% in male and in female both. The difference between male and female hand ratio was statistically significant in the study by Rossiter, while it was not significant in our study.

Moreover, the palm ratio in adults male and female was 0.49% and 0.51%, respectively, with significant difference between male and female. Rossiter[10] also found the significant difference in the palm ratio between male and female.

In our study, the hand ratio in children was 1.065% with no significant difference between male and female as compared to 0.94% by Nagel[11] and 0.82% by Perry[9] and 0.87% in the study by Amirsheybani.[6] The palm ratio in children was 0.635% in our study with no significant difference between male and female as compared to 0.52% by Nagel[11] and 0.45% in the study by Perry.[9]

Amirsheybani[6] used integrated planimeter to calculate hand and palm surface area from 800 Caucasian volunteers ranged in age from 2 to 89 years. The BSA was determined by using Dubois's and Gehan and George's formula. The palmar surface of the hand corresponds to 0.78 ± 0.08 percent of the BSA in adults. In children the palmar surface of the hand was 0.87 ± 0.06.

Perry[9] studied 20 adult and 10 children (age undocumented). BSA was calculated by the method of Gehan and George. The area of hand projection was determined using the computer program. Among the adults the means of palm and whole hand surface area with 95% confidence intervals were 0.41% (0.37 to 0.43) and 0.77% (0.74 to 0.80) respectively. Among the children the corresponding values were 0.45% (0.42 to 0.48%) and 0.82% (0.78 to 0.87%). For the two groups combined the mean projected whole hand area was 0.79% (0.76 to 0 .81%).

Rossiter[10] also published a study of 70 (36 male and 34 female) adult subject in which TBSA was calculated by standard nomogram and hand surface area was calculated using hand outline drawn on a piece of graph paper. The areas were calculated by counting the squares enclosed in the outlines. They found that the average area of palm was 0.52% and 0.43% of TBSA in males and females, respectively. The average area of hand was 0.81% in males and 0.67% in females.

Nagel[11] calculated the TBSA by standard nomogram and hand and palm surface area was calculated using photocopy of the hand. They found that in 91 children the average area of hand was 0.94% (95% CI) and the area of palm was 0.52% (95% CI) of TBSA.

Sheridan[12] measured the palmar surface of the hand in 69 patients. They concluded that the surface of the palm averaged 0.52% of TBSA and the palmar surface the hand 0.85% of TBSA. However, they did not find any correlation with age or sex.

With this study it is evident that using hand surface area alone, the size of the burn will be overestimated. For more accurate assessment the resultant area should be multiplied by 0.9 for adults. We have used the simple tracing of hand for determination of hand/ palm surface area because it is a simple and convenient method and it is within 2% of the hand area measured by an integrated planimeter.[6] Therefore one may not need an integrated planimeter, computer assisted methods or sophisticated scale to measure hand area. We have also used dominant hand tracing for area measurement because there is no significant difference between the areas of two hands.[6] In children the age ranged from 2-17 years because studies have shown that the hand area maintains a fairly constant percentage of BSA throughout the process of growth in the same age range.[6] The limitation of the study is that estimation of hand/palm surface area and BSA is calculated using height and weight nomogram and indirectly related to the accuracy of formula and possibility of subjective errors. Human body is three dimensional and currently no practical method is available to measure three dimensional surface area of the body. Using patients hand surface area is a simple method of assessing the size of burn or injury especially in small patchy burn and in extensive burns where non burned area can be counted using hand. The results indicate that whole hand not the palm represents more closely to 1% of TBSA.

## CONCLUSION

Mean hand area in adult was 139.462 ± 16.21 cm^2^ and the same for child was 85.646 ± 21.11 cm^2^. 0 Mean palm area in adult and children was 75.756 ± 9.938 cm^2^ and 50.675 ± 12.603 cm^2^, respectively. In our study, the mean hand ratio in adult was 0.921 ± 0.088 and for children it was 1.065 ± 0.110. In many studies done in western countries, the ratio of hand area to TBSA is found to be around 0.8%. This may be due to reduced TBSA of Indian population as compared to their western counterpart. Mean palm ratio in adult was 0.502 ± 0.065 and in child was 0.632 ± 0.084. The palm ratio in western population for adult and children was 0.4% and 0.5%, respectively. We can observe that the results of our study differ with that of western studies. The hand area as compared to TBSA more closely represents 1% of TBSA in Indian population.
